# Estimation of the Vehicle Speed Using Cross-Correlation Algorithms and MEMS Wireless Sensors

**DOI:** 10.3390/s21051721

**Published:** 2021-03-02

**Authors:** Cheng Zhang, Shihui Shen, Hai Huang, Linbing Wang

**Affiliations:** 1 Department of Civil and Environmental Engineering, Pennsylvania State University, State College, PA 16801, USA; c.zhang@psu.edu; 2 Rail Transportation Engineering, Penn State Altoona, Altoona, PA 16601, USA; huh16@psu.edu; 3 Department of Civil and Environmental Engineering, Virginia Tech University, Blacksburg, VA 24060, USA; wangl@vt.edu

**Keywords:** speed estimation, micro-electromechanical sensor, wireless sensor, cross-correlation, accelerated pavement testing

## Abstract

Traffic information is critical for pavement design, management, and health monitoring. Numerous in-pavement sensors have been developed and installed to collect the traffic volume and loading amplitude. However, limited attention has been paid to the algorithm of vehicle speed estimation. This research focuses on the estimation of the vehicle speed based on a cross-correlation method. A novel wireless micro-electromechanical sensor (MEMS), Smartrock is used to capture the triaxial acceleration, rotation, and stress data. The cross-correlation algorithms, i.e., normalized cross-correlation (NCC) algorithm, the smoothed coherence transform (SCOT) algorithm, and the phase transform (PHAT) algorithm, are applied to estimate the loading speed of an accelerated pavement test (APT) and the traffic speed in the field. The signal-noise-ratio (SNR) and the mean relative error (MRE) are utilized to evaluate the stability and accuracy of the algorithms. The results show that both the correlated noise and independent noise have significant influence in the field data. The SCOT algorithm is recommended for speed estimation with reasonable accuracy and stability because of a large SNR value and the lowest MRE value among the algorithms. The loading speed investigated in this study was within 50 km/h and further verification is needed for higher speed estimation.

## 1. Introduction

Traffic information, including but not limited to traffic volume, vehicle speed, and axle load, is one of the most important input factors for pavement design, management, and health monitoring. It also has a crucial influence on the performance of asphalt pavement. In the Superpave mix design, the performance grade (PG) of asphalt is selected based on the traffic loading rate and equivalent single axle loads (ESALs) that is calculated by the traffic volume and axle weight [1]. The mechanistic-empirical pavement design (MEPDG) requires ESALs, wheel wander, wheelbase distribution, traffic load rate, and the like [2].

Many studies have concentrated on obtaining the vehicle classification and the axel load and improving the monitoring accuracy. For example, weigh in motion (WIM) is one of the most widely used monitoring systems in the US for collecting traffic information. After decades of development, WIM can provide various traffic information, such as classification of vehicles, total gross weight and axle weight, date, and time [3]. Hernandez [3] integrated inductive loop detectors into the WIM system and a truck body class model was developed to classify trucks with the multiple classifier system approaches. According to the field test results, the multiple classifier system approach improved the classification accuracy. The overall correct classification rate was above 80% with only two of the 54 body classes possessing the correct classification rate below 60%. Birgin [4] combined a piezoresistive material with a cement concrete to develop a novel material. This material paved on the bridge was utilized as the sensors in the WIM system to determine the vehicle classification and the vehicle weight [4]. Such research contributed to improving the sensor technology of the WIM system and enhancing the accuracy of classification.

The speed can be calculated with the distance between two sensors divided by the time delay between the two signals. The time delay is a critical parameter to determine the speed since the distance can be measured directly. Many researchers have applied anisotropic magnetoresistive (AMR) sensors to obtain the time delay by detecting the change of the magnetic field [5]. Zhu [6] illustrated the cross-correlation method with the AMR sensors for vehicle parking detection and vehicle speed estimation. Taghvaeeyan [7] described a circular convolution algorithm that can improve the calculation efficiency of the cross-correlation. The cross-correlation method was adopted to calculate the time delay. Markevicius et al. [8] introduced and compared four methods to estimate the time delay for calculating the vehicle speed based on an AMR system. The results indicated that the cross-correlation based algorithms are more accurate than the other two methods that calculate the sum of absolute differences and the difference in gravity (mass) centers. However, it is also noted by the researchers [8] that the AMR sensor can be influenced by environment temperature and any massive metal objects in the area of the sensor.

The Micro-Electromechanical Sensor (MEMS) has been developed in many areas including the medical, the automotive industries, and civil engineering [9,10]. Advancements in MEMS technology and wireless sensor networks provide a non-destructive monitoring method [11] for long-term, continuous, real-time structural health monitoring of pavement at low cost within the context of sustainable infrastructure systems [10,12]. The MEMS can be convenient to install and can protect the researchers from a dangerous situation through remote monitoring and controlling. The in-pavement MEMS can also monitor the mechanical response of the pavement. Comparing with the conventional AMR sensor system and the WIM system, the MEMS is more versatile due to its collective sensing units included. The mechanical properties of the pavement during construction and service time as well as the traffic information can be estimated using one single system, which is convenient and cost effective [13,14,15].

This paper aims to estimate the vehicle speed based on a cross-correlation method and a novel MEMS, SmartRock sensor. SmartRock sensors were installed in an asphalt pavement test section using an accelerated pavement testing (APT) loading facility as well as an in-situ pavement to collect the pavement responses. The vertical stress response was used to estimate the speed with three different cross-correlation algorithms. The noises of the data were analyzed based on the algorithms and the most effective cross-correlation algorithm was recommended.

## 2. MEMS SmartRock Sensor

**Figure 1:**
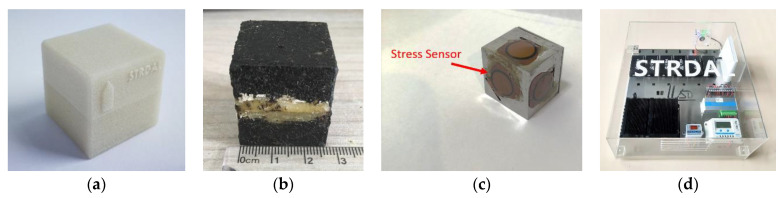
SmartRock sensors: (**a**) 3D printed SmartRock; (**b**) size of SmartRock; (**c**) Smartrock with stress sensors; (**d**) wireless data acquisition (DAQ).

SmartRock (Figure 1) is a wireless particle motion sensor. The small-size version (Cube with side length 27 mm) of SmartRock is close to the size of a coarse aggregate in the pavement. The 3D printed external shell of SmartRock uses high-temperature-resistant thermoplastic polymer materials [15] that can survive in the paving and compaction environment. Thus, SmartRock can be conveniently installed during the laydown of the hot mixture. Wang et al. [15] showed that the SmartRock can survive in the high-temperature environment (140–170 °C) under both laboratory and field compaction. The internal measuring units of the Smartrock can record real-time tri-axial acceleration, tri-axial rotation, and three-dimensional surface stress, temperature, and time. More details and specifications of the sensor are listed in Table 1. The data are transmitted and saved in the local adapter system via Bluetooth low energy (BLE) technology. The SmartRock has a low energy consuming mode (sleeping capability); it can be put into sleep or waken up according to the monitoring requirement for saving battery time. The microcontroller unit (MCU) within SmartRock can check and optimize the data in real-time to realize “self-adaptation” (defined as the abilities of automatic perception, self-learning, and real-time processing). By combining the collected data with computer simulations using such as the finite element method or the discrete element, SmartRock sensing technology can achieve more accurate simulation based on sensing mechanism and real time (SMART) computing algorithm [16].

SmartRock sensors have been utilized to quantify the ballast particle movement, including the peak of vertical and horizontal acceleration, and the angular acceleration during ballast deformation in the railroad [17]. They have also been used to monitor the compaction process of the asphalt pavement by tracing the particle movement characteristics including both rotation and acceleration [15]. SmartRock has prominent advantages in convenience, durability, reusability, and stability in contrast to traditional sensors. Therefore, the SmartRock monitoring system was selected in this study to collect data.

## 3. Calibration of Stress Measurement

**Figure 2:**
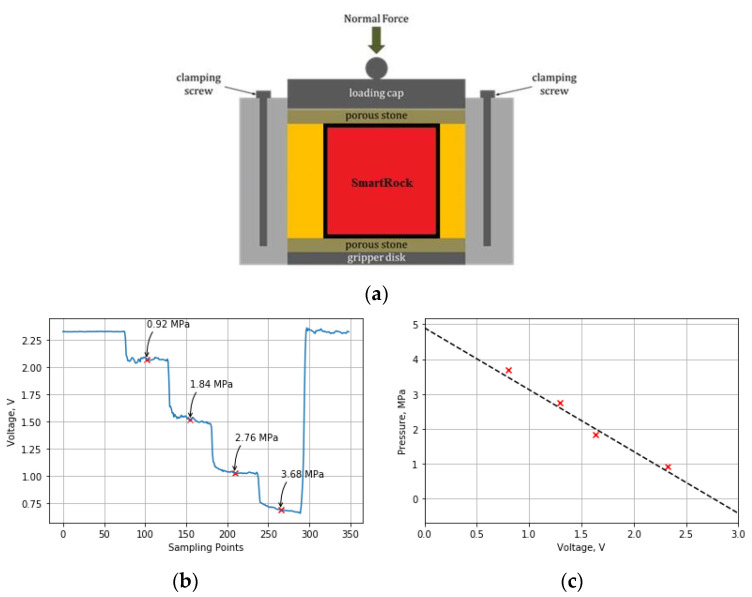
Calibration of the stress cell of SmartRock: (**a**) Equipment of the direct shear test; (**b**) Voltage signal under pressure; (**c**) Relationship between voltage and stress.

The three-dimensional stress is recorded by the stress gauge within SmartRock. The output of the stress gauge is the voltage signal. At the same temperature, the voltage has a negative linear relationship with the pressure, as expressed in Equation (1).

(1) $$\sigma =\frac{a\left(U_{0}-U\right)-b\cdot \ln\left(T\right)+c}{A}$$

where σ is the pressure; *U*_0_ is the initial voltage before loading; *U* is the measured voltage under loading; *T* is the temperature; *A* is the area of the stress gauge; *a*, *b*, and *c* are parameters that should be calibrated before installation. In this study, the SmartRocks were calibrated using a direct shear test loading setup, as shown in Figure 2a. The different static normal forces were applied to the SmartRock by the loading cap. The voltage signals were recorded as shown in Figure 2b. The mean values of voltage signals were calculated for each stress. The parameters, a, b, and c can be regressed with the recorded signals and the normal forces as shown in Figure 2c. After calibration, a verification was conducted using the same equipment. The coefficient of variation (CV) for the verification data is 4.5%. In other words, the contact stress applied on the SmartRock can be reasonably determined by the voltage signals using Equation (1).

## 4. Cross-Correlation Method

The cross-correlation method has been used in motion estimation [18], health assessment [19], and the passive SONAR array system [20]. Various algorithms have been developed to estimate the time delay for different signals based on the cross-correlation method. Knapp [21] discussed the commonly used algorithm such as Roth Impulse Response, the Smoothed Coherence Transform (SCOT), the Phase Transform (PHAT), Eckart, and so on. In this paper, three algorithms of the cross-correlation method are described and used to calculate loading speed.

The cross-correlation that is the overlapped area of two signals can indicate the similarity of the different signals, as shown in Equation (2):

(2) $$R_{xy}=\int_{-\infty }^{+\infty }f\left(t\right)g\left(t+\tau \right)dt,$$

where, $R_{xy}$ is the cross-correlation. f(t) and g(t+τ) are signals. *τ* is the lag. From Equation (2), the cross-correlation can be calculated at different lags *τ*. The maximum value of the cross-correlation can be found at a certain lag *τ* that is the time delay between the two signals.

In the signal generation and propagation, it is unavoidable that the signal is mixed with noises that have a significant influence on the cross-correlation results. So various algorithms were developed to reduce the effects of noises. The collected data can be assumed to consist of the signal and the noise, as shown in Equations (3) and (4):

(3) $$f\left(t\right)=s_{1}\left(t\right)+n_{1}\left(t\right),$$

(4) $$g\left(t\right)=s_{2}\left(t\right)+n_{2}\left(t\right),$$

where $s_{1}\left(t\right)\;\mathrm{and}\;s_{2}\left(t\right)$ are the signals; $n_{1}\left(t\right)$ and $n_{2}\left(t\right)$ are the noises. If $n_{1}\left(t\right)$ and $n_{2}\left(t\right)$ are independent, the $n_{1}\left(t\right)$ and $n_{2}\left(t\right)$ are the independent noises. Otherwise, they are correlated noises. The SCOT algorithm and The PHAT algorithm are applied in this study to remove the correlated and independent noises and obtain an accurate cross-correlation calculation.

The stability and accuracy of the cross-correlation algorithms will be evaluated based on the signal-noise-ratio (SNR) and the mean relative error (MRE). The SNR can be defined as Equation (5) [22]:

(5) $$SNR=\frac{A^{2}}{2\sigma^{2}},$$

where A and σ are the amplitude and standard deviation of the data. The SNR will increase with the noise components decreasing. For the cross-correlation algorithm, the higher SNR is, the more noises are filtered, and the better stability of the algorithm has. The MRE can be calculated by Equation (6):

(6) $$MRE=\frac{1}{n}\sum \frac{\left|U-V\right|}{V},$$

where *U* is the estimated velocity, and *V* is the measured velocity. *A* smaller number of MRE indicates a higher accuracy of the estimation. Comparing with the conventional statistical parameters like R^2^, root mean square error (RMSE) and mean absolute error (MAE), MRE is more suitable to evaluate the performance of different algorithms because it can reduce the influence from the magnitude of the measured values.

### 4.1. The Normalized Cross-Correlation (NCC) Algorithm

In practice, the NCC algorithm (Equation (7)) is a widely used cross-correlation algorithm without considering noises. The main advantage of the NCC is that it is less sensitive to linear changes in the amplitude of signals [23]:

(7) $$NCC\left(\tau \right)=\frac{R_{xy}\left(\tau \right)}{\sqrt{\sigma_{x}\sigma_{y}}},$$

where: $\sigma_{x}$ and $\sigma_{y}$ are the standard deviation of the signal f(t) and signal g(t).

### 4.2. The SCOT Algorithm

The SCOT algorithm was developed to determine time delays between weak broad-band correlated noises received at two sensors [24]:

(8) $$R_{xy}\left(\tau \right)=\int_{-\infty }^{+\infty }G_{xy}\left(f\right)w\left(f\right)e^{2i\pi f\tau }df,$$

where:

(9) $$w\left(f\right)=\frac{G_{xy}\left(f\right)}{\sqrt{G_{x}\left(f\right)G_{y}\left(f\right)}},$$

$G_{x}\left(f\right)$ and $G_{y}\left(f\right)$ are the auto spectra of signals *x*(t) and *y*(t). $G_{xy}\left(f\right)$ is the cross spectra. The correlated noises can be filtered by this algorithm. Furthermore, if the signal-noise-ratio (SNR) of the SCOT results are larger than the SNR of the NCC results, the correlated noise should be considered as a part of noises. However, when the noises are independent, the weight function (Equation (9)) exhibits spreading. So, the SCOT algorithm can be used to calculate the cross-correlation of the signals with the correlated noises.

### 4.3. The PHAT Algorithm

For the independent noises, the PHAT algorithm can be used to avoid spreading. The calculation process is the same as the SCOT algorithm, while the weight function is defined as shown in Equation (10):

(10) $$w\left(f\right)=\frac{1}{\left|G_{xy}\left(f\right)\right|},$$

Same to the SCOT algorithm, if the SNR of the PHAT results is larger than that of the NCC results, the independent noise should be considered. In addition, the type of noise can be decided by comparing the SNR of the SCOT algorithm and the PHAT algorithm.

In a summary, the SCOT algorithm and the PHAT algorithm can remove the correlated and independent noises, respectively. The type of noise in the pavement can also be recognized by comparing the results of both algorithms.

## 5. Experimental Configurations

In this study, two experimental configurations were established to implement the idea of using SmartRock sensing data and cross-correlation method to estimate vehicle speed. One experiment was conducted at a laboratory test section with accelerated pavement testing (APT) facility, and the other one was performed at an in-situ pavement.

**Figure 3:**
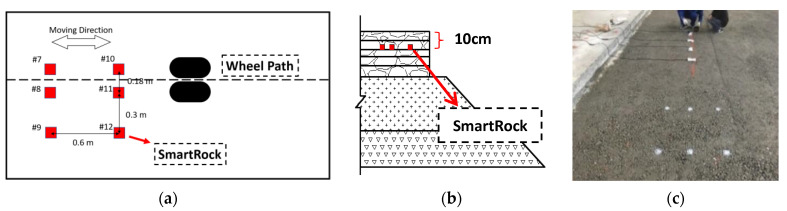
The configuration of the APT test: (**a**) Top view; (**b**) Side view; (**c**) The embedded SmartRocks.

The APT equipment was a Mobile Loading Simulator 66 (MLS66). The SmartRock monitoring system was installed in the full-scale APT pavement section (Figure 3). Two groups of SmartRock sensors were embedded. One group was embedded under the wheel path and the other was embedded 30 cm away from the outside of the wheel path. The pavement structure consisted of four layers: HMA surface layer, semi-rigid base, graded aggregate subbase, and subgrade. The HMA surface layer was 25-cm AC-13 asphalt mixture, which was compacted in five lifts: 5 cm, 5 cm, 4 cm, 5 cm, and 6 cm. The SmartRock Sensors were all buried in the 10-cm depth of the surface layer (bottom of the second lift). This testing was conducted at a controlled temperature of 24 °C. The loading speeds were 5 km/h, 10 km/h, 15 km/h, and 20 km/h. At each speed, five dynamic loading amplitudes were applied including 20 KN, 35 KN, 50 KN, 60 KN, and 75 KN respectively, and the data collection frequency was 100 Hz. Multiple SmartRocks were installed for different research purposes. #8 and #11 were used to collect the data during compaction process. #9 and #12 were used to analyze the wandering effects. #7 and #10 were designated for the speed estimation.

**Figure 4:**
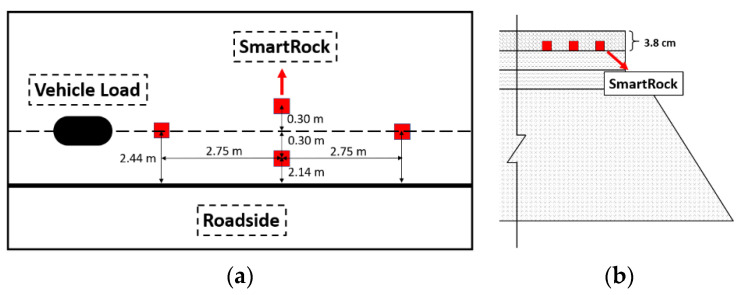
The configuration of the Field test: (**a**) Top view; (**b**) Side view.

The field test was a pavement maintenance project located in Altoona, Pennsylvania. The original pavement surface was milled out by 6.4 cm, followed by two layers of overlay (a 2.6-cm leveling courses plus a 3.8-cm wearing course). The wearing course mixture is a PG 64E-22 warm mix asphalt (WMA) mixture with nominal maximum aggregate size of 9.5 mm. The design traffic level is 0.3 to 3 million equivalent single axle loads (ESALs). Figure 4 shows the SmartRock configuration in the pavement. Four SmartRock sensors (#2, #3, #5, #6) were embedded at the bottom of the wearing course during the construction, with two (#3 and #5) at the wheel path and two (#2 and #6) at 30 cm away from the wheel path. After opening traffic, SmartRock collected 30 min of stress data at 16:00 every Wednesday from 12 August 2020 to 23 September 2020. The temperature in the pavement ranges from 31 °C to 48 °C. At the same time, a speed radar was used to measure the vehicle speed as a reference.

## 6. Results and Discussions

The stress data collected from the APT test and the field pavement were used to estimate the loading speed.

### 6.1. Stress Data Analysis

**Figure 5:**
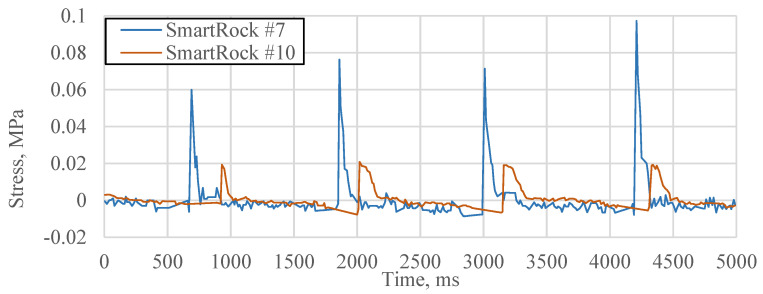
An example of the collected vertical stress.

The vertical stress signals collected by two Smartrocks were utilized to calculate the loading speed. In the APT test, the distance between the two Smartrocks is 0.6 m. Figure 5 shows a section of the stress signals (under the 50 kN, 15 km/h loads) extracted from the two SmartRocks. The Sampling frequency is 100 Hz. When the load is approaching and leaving, the vertical stress rapidly increases to the peak value followed by a stress recovery. The data collected from different SmartRocks can be separated and matched directly, since the loading velocity and the wheel path were fixed.

**Figure 6:**
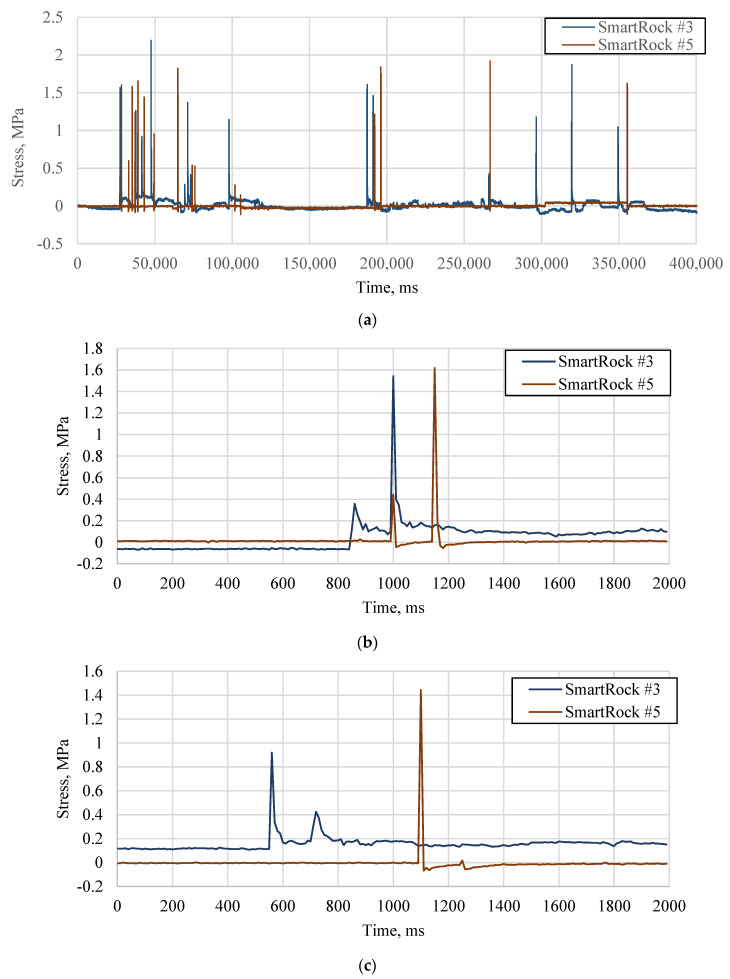

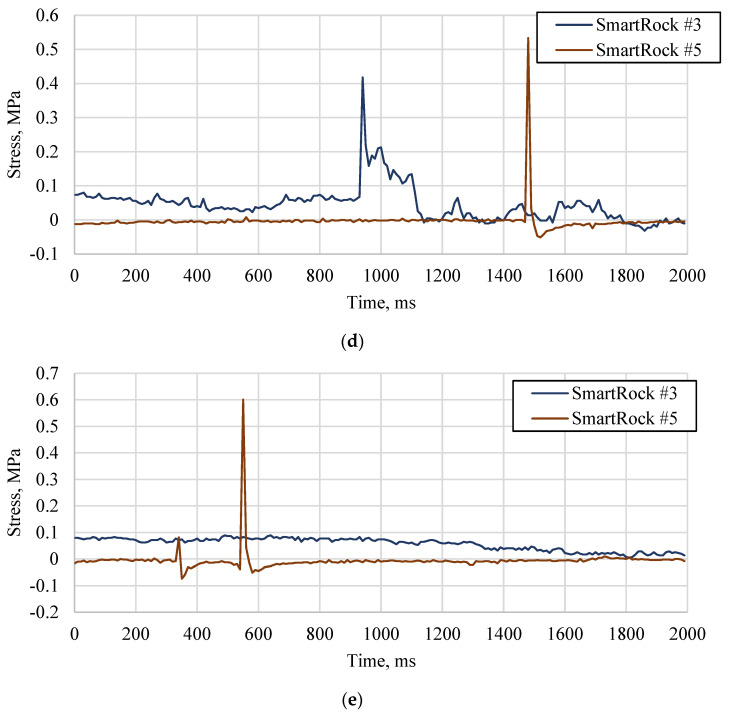
Collected Stress Signals in the Field (**a**) a series of stress signal; (**b**) case 1: two SmartRocks captured two axle loads, respectively; (**c**) case 2: one of the SmartRocks only captured an axle load; (**d**) case 3: two SmartRocks captured an axle load, respectively; (**e**) case 4: one of the SmartRock didn’t capture the signals.

In the field pavement, the distance between the two SmartRocks is 5.5 m. A series of signals under the vehicle loads were extracted from SmartRocks #3 and #5 (Figure 6a). It is found that although there are different scenarios when the signals are very different (Figure 6b–e), the trend of individual signal is similar to the signals collected from the APT test. Most cases belong to case 1, of which both two axle loads of a vehicle were captured by two SmartRocks, and the time delay can be accurately calculated using the cross-correlation method, as shown in Figure 6b. In case 2 (Figure 6c), one of the SmartRocks (For example SmartRock #5) only captured an axle load of the vehicle, while the SmartRock #3 captured two axle loads. Case 3 (Figure 6d) shows that two SmartRocks captured one axle load, respectively. These phenomena made it difficult to determine the specific axle load that caused the corresponding peak stress and could result in theoretical errors in the case 2 and case 3. Figure 6e shows that only one SmartRock captured a vehicle load. In this case, the data were removed in the calculation because the time delay is hard to be obtained from an individual SmartRock.

In the field, matching the collected data is more complicated due to more sophisticate traffic environment (traffic grouping, wandering, noises, etc.). Several strategies were used to solve this problem. First, only the peaks greater than the three-sigma limit were selected as the effect of vehicle loads; others were treated as noises [25]. In addition, to prevent mixing stress responses from two different vehicles selected for cross-correlation analysis, some constraints were added. If the time interval of two stress responses is greater than 1 s, it is believed that these two responses belong to two vehicles given the regular vehicle speed of 10–60 mph and the typical axle distance of 2.34 m to 4.32 m. Finally, the timestamps recorded by SmartRocks were introduced to ensure the stress signals collected by the two SmartRocks were actually caused by the same vehicle.

### 6.2. Calculation of Vehicle Speed

**Figure 7:**
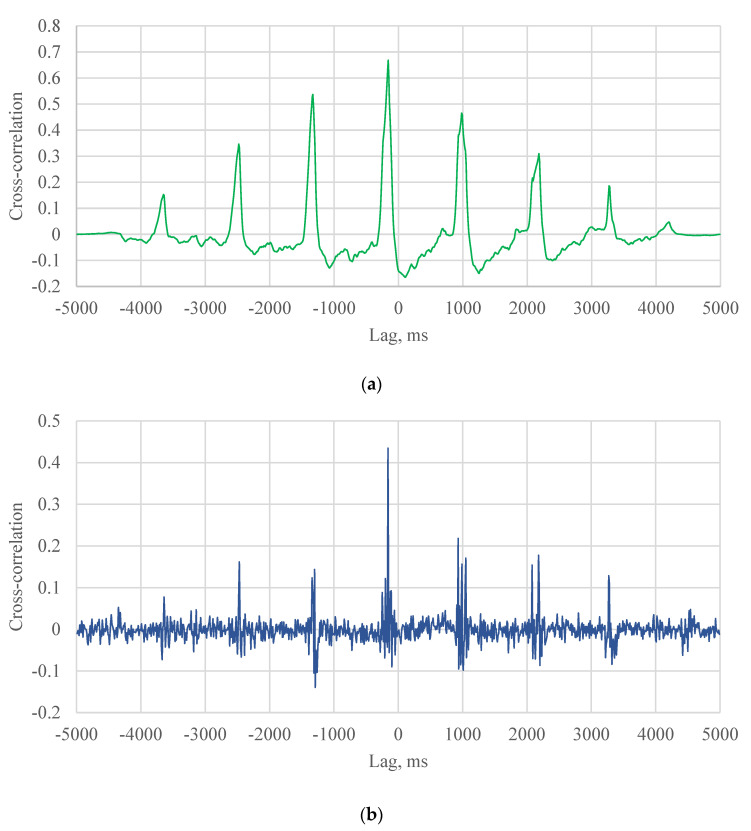

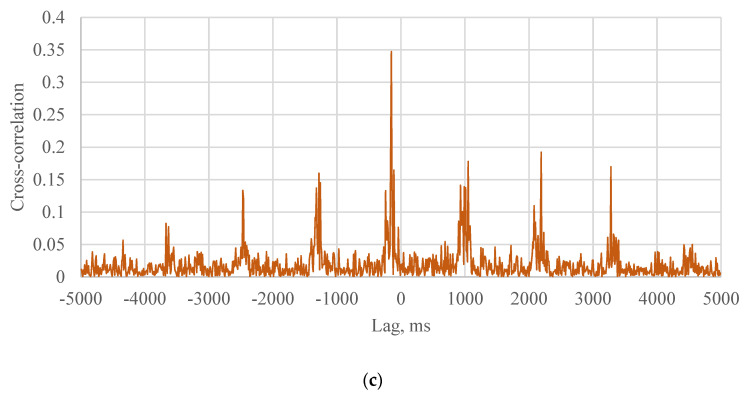
An example of the cross-correlation results for APT test: (**a**) The NCC algorithm, (**b**) The PHAT algorithm, (**c**) The SCOT algorithm.

The cross-correlation results of APT test for different algorithms (the NCC, the SCOT, the PHAT) are shown in Figure 7. These figures show a series of peaks. The time delay is the lag location of the maximum cross-correlation value at which the two signals are matched. No wandering effect is considered for the APT loading. When the lag is equal to the time delay plus another cycle period, the cross-correlation also shows a peak value. These peaks are considered as noises because the two signals are not matched at these lag locations.

**Figure 8:**
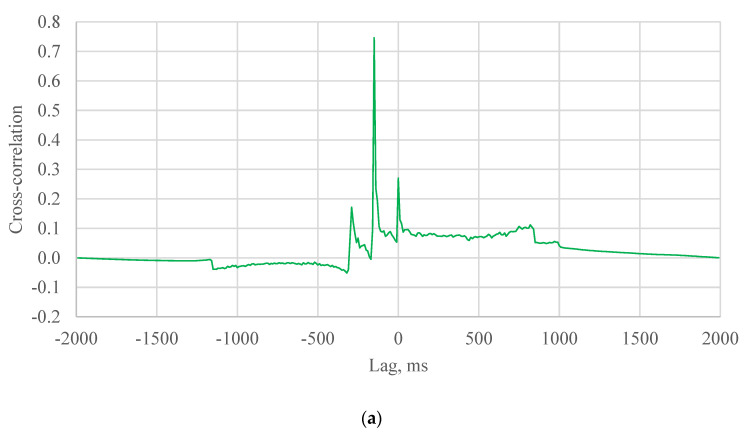

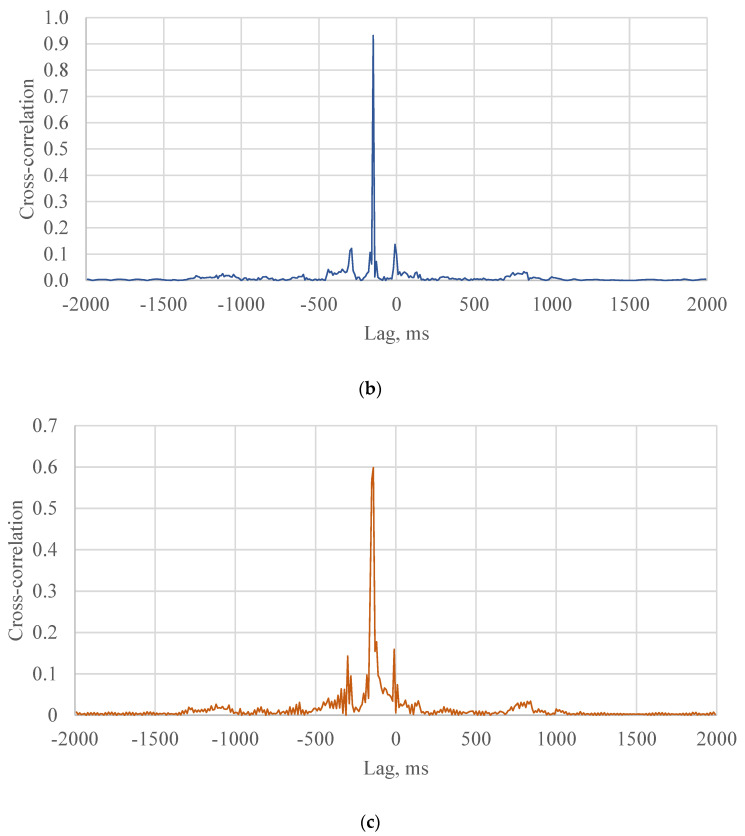
An example of the cross-correlation results for field test: (**a**) The NCC algorithm, (**b**) The PHAT algorithm, (**c**) The SCOT algorithm.

**Figure 9:**
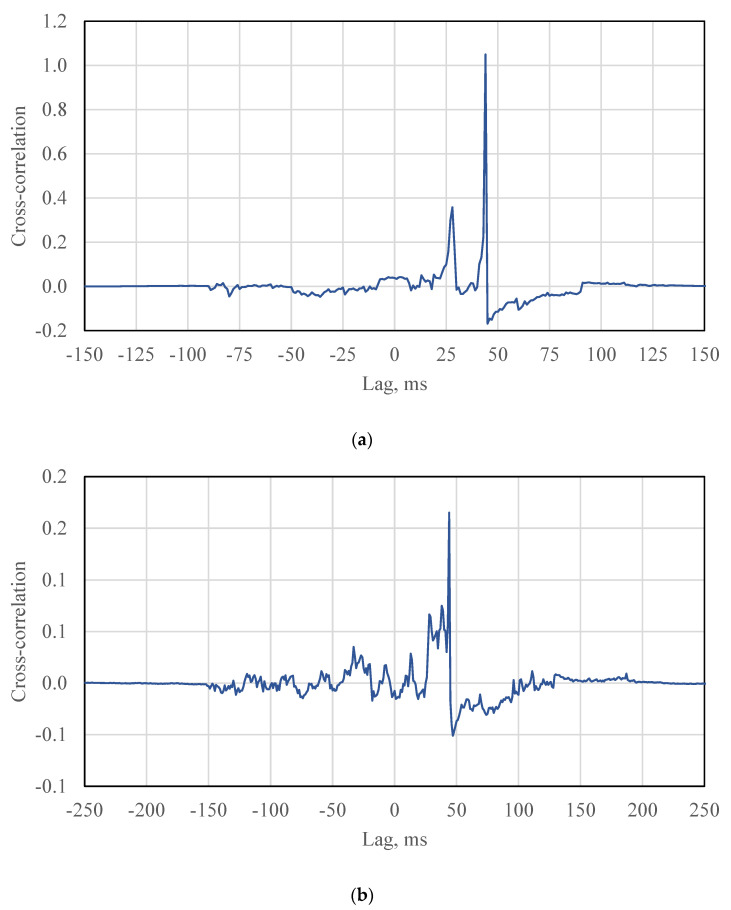
An example of the cross-correlation results (**a**) case 2: one of the SmartRocks only captured an axle load; (**b**) case 3: two SmartRocks captured an axle load, respectively.

In the field, there are few peaks of cross-correlation for each vehicle (Figure 8 and Figure 9). The time delay is supposed to be the lag location of the maximum cross-correlation value. the speed can hence be estimated using the determined time delay and the distance between two SmartRock sensors (5.5 m in the field test).

For different cases in the field test, the hypothesis that the time delay is the lag of the maximum cross-correlation value may not always be true and must be evaluated according the specific scenarios. The probability that the hypothesis can be accepted should be calculated. The SCOT was taken as an example of algorithms to analyze the hypothesis. In case 1 that the two axle loads were captured by two SmartRocks, three peaks were shown in the cross-correlation result, as shown in the Figure 8c. If the two signals were matched, the maximum cross-correlation should be the second peak value. In other words, the hypothesis can be accepted in the case 1.

In case 2, one of axle loads was missed by SmarRock #5 (Figure 6c). Two peaks were shown in the cross-correlation result (Figure 9a). There are four situations. The SmarRock #5 captured the front axle load that matched with the front axle load captured by SmarRock #3. In this situation, the maximum cross-correlation should be the first peak value. The hypothesis can be accepted. Or the SmarRock #5 captured the front axle load that matched with the rear axle load captured by SmarRock #3. The maximum cross-correlation should be the second peak value. The hypothesis should be rejected. The other situations can be analyzed in the same way. Therefore, the probability of accepting the hypothesis should be 50% for the case 2.

Case 3 is that two SmartRocks captured one axle load respectively (Figure 6d). There is only one peak in the cross-correlation result (Figure 9b). There are also four situations. The analysis approach is similar to the case 2. In summary, the probability of accepting the hypothesis should also be 50% for case 3.

From the field test data, most of the collected data belonged to the case 1 accounting for 72.3% of the total cases. The proportions of case 2 and case 3 are 17.0% and 13.6% respectively. According to the law of total probability [26], the probability that the time delay is the lag location of the maximum cross-correlation value is 84.7%. In other words, the number of the matched cases is 84.7% of the total quantity. 15.3% of the results contained theoretical errors that contributed to the RE values as presented later in this paper.

**Figure 10:**
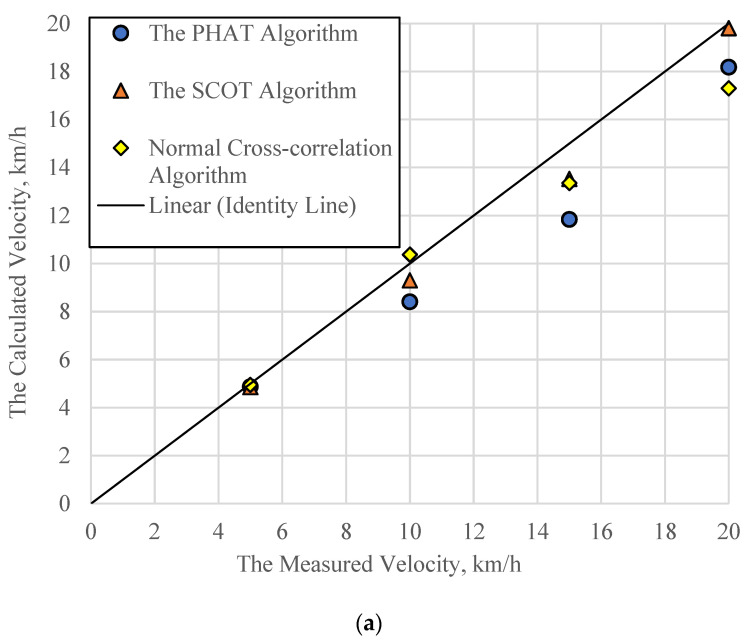

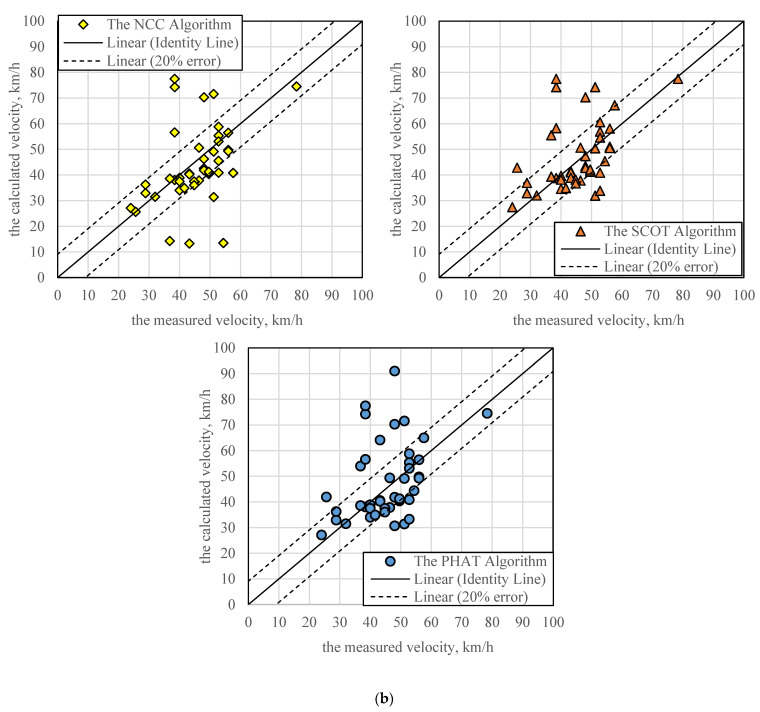
The calculated velocity: (**a**) The APT test results, (**b**) The field test results.

Figure 10 presents a comparison between the estimated speeds using different cross-correlation algorithms and the measured speeds, illustrated in a line of equality plot. A set of 20% error lines were also included in the figure to show the prediction quality. In general, most data points are within the 20% error lines and the three algorithms seem to give reasonable estimation for the speeds. Large errors do happen in some cases which may be related to the data capturing capability of the existing sensor setup under sophisticated traffic conditions. In addition, the SCOT algorithm has the least points beyond the 20% error zone, indicating it might be the most promising algorithm among the three. A quantitative error analysis of these three algorithms will be discussed in the next section.

### 6.3. Reliability of Cross-Correlation Algorithms

The SNR and MRE values are calculated to evaluate the reliability of the three cross-correlation algorithms. For the APT test, because the SNR and the MRE values are relatively consistent for each algorithm, only the average results from one loading amplitude of 50 kN but varying speeds from 5 km/h to 20 km/h are included in Table 2. The SNR and the MRE values of the field data are listed in Table 3. It is found that the PHAT and the SCOT are more stable than the NCC algorithm, given their relatively high SNR value. The high SNR values indicate that the correlated and the independent noises are removed by the SCOT and the PHAT, respectively. In other words, the collected vertical stress data include correlated and independent noises. Besides, in the APT test, the SNR value of the SCOT is close to the SNR value of the PHAT. In the field test, the SNR value of the PHAT is much larger than that of the SCOT. It indicates that there are more independent noises in the field test. Considering the difference between the APT test and the field test, the vehicles on the other lanes could be a part of the sources of the independent noises.

Furthermore, the SCOT algorithm, compared with the PHAT algorithm, has better accuracy due to its low MRE values. This indicates that the main parts of noises are correlated noise that could come from the test procedure (like the vibration of wheel loads, temperature change, etc.). The variation of SNR and MRE for the field data are higher than those of the APT data. This high variation can be related to the variation of the vehicle speeds in the testing section and the measurement error of speed radar. Above all, the cross-correlation method can be used to estimate vehicle speed with reasonable stability and accuracy. The SCOT algorithm is recommended to analyze the test data.

## 7. Conclusions and Recommendation for Future Work

This paper focuses on the estimation of the vehicle speed with the cross-correlation methods based on in-pavement MEMS sensors. Three cross-correlation algorithms, the NCC algorithm, the SCOT algorithm, and the PHAT algorithm were introduced. The vertical stress data were collected by SmartRock sensors in the APT test and the in-situ pavement to estimate the loading speed.

The following conclusions and recommendations for future work can be obtained.

- SmartRock sensor, a type of MEMS wireless sensors, when placed in series in the pavement, can be used to reasonably estimate the speed of the loading vehicles based on measured stress signals. In this study, the distances between two SmartRocks are 0.6 m in the APT test, and 5.5 m in the field test. These distances are acceptable for the 20 km/h and 50 km/h. For future research and applications, it is recommended that a suitable distance for the higher speed need to be tested, and the influence of the distance on the estimation accuracy should be further evaluated.
- The cross-correlation method can be used to estimate the loading speed. The SCOT algorithm is recommended to estimate the vehicle speed due to its relatively high SNR values and low MRE values. The high SNR value indicates that the SCOT algorithm has better stability and is hardly influenced by the abnormal values. The low MRE value indicates it has better accuracy for the speed estimation.
- Based on the configuration of the field sensors, the number of cases in which the captured signals can be matched is 84.7% of the total number of cases. On the other hand, 15.3% of the results contained theoretical errors that contributed to MRE values. Missing axle loads is the source of the theoretical error. Therefore, increasing the ability to capture axle loads will improve the estimation accuracy. In addition, the configuration of the sensor installation in the field and the analysis algorithms need to be further researched to improve the data capturing capability and the estimation accuracy.
- The signals of the vertical stress are mixed with correlated noises and independent noises. The correlated noises are the main noises in the APT test, while in the field the independent noises are the main noises that have little influence on the speed estimation. A part of the independent noises could be caused by the vehicles on the other lanes. However, more tests are needed to prove this hypothesis.
- The cross-correlation algorithms along with the embedded SmartRock wireless sensors are found to be a promising method for estimating the vehicles’ speed in the field. It is worth noting that the loading speed investigated in this study was 5 km/h to 20 km/h in the APT test, and 15 km/h to 50 km/h in the field test. The accuracy of estimation for higher speeds needs further evaluation.

## Figures and Tables

**Table 1:** Specifications of Smartrock.

Items	Parameters
Size	27 × 27 × 27 mm
Stress range	1–100 lbs
Accelerometer	±2/±4/±8/±16 g
Gyroscope	±250/±500/±1000/±2000 o/s
Magnetometer	±4800 uT, 16 bits
Power consumption	Working current 10 mA; Waiting Current 0.4 mA; Sleeping Current 0.012 mA
Power supply	high temperature resistant lithium manganese battery, 3V@350 mAh
Communication	Bluetooth 4.0 BLE, 2.412~2.484 GHz
Communication Distance	5–20 m

**Table 2:** The comparison of the cross-correlation algorithm with the APT data.

Index	NCC Algorithm	SCOT Algorithm	PHAT Algorithm
SNR	5.90 ± 0.33	12.98 ± 0.75	13.76 ± 0.96
MRE	0.073 ±0.018	0.052 ± 0.012	0.122 ± 0.024

**Table 3:** The comparison of the cross-correlation algorithm with the field data.

Index	NCC Algorithm	SCOT Algorithm	PHAT Algorithm
SNR	66.75 ± 46.01	72.04 ± 25.65	127.89 ± 41.12
MRE	0.29 ± 0.33	0.22 ± 0.24	0.25 ± 0.25

## Data Availability

All data generated or analyzed during this study are included in this published article.
